# Lifetime of *Para*hydrogen in Aqueous Solutions and Human Blood

**DOI:** 10.1002/cphc.201900670

**Published:** 2019-09-12

**Authors:** Andreas B. Schmidt, Jakob Wörner, Andrey Pravdivtsev, Stephan Knecht, Harald Scherer, Stefan Weber, Jürgen Hennig, Dominik von Elverfeldt, Jan‐Bernd Hövener

**Affiliations:** ^1^ Department of Radiology, Medical Physics Albert-Ludwigs-Universität Freiburg Universitätsklinikum Freiburg Kilianstr. 5A 79106 Freiburg Germany.; ^2^ Department of Radiology and Neuroradiology Section Biomedical Imaging, MOIN CC Universitätsklinikum Schleswig-Holstein, Universität Kiel Am Botanischen Garten 14 24118 Kiel Germany; ^3^ Institut für Physikalische Chemie Albert-Ludwigs-Universität Freiburg Albertstr. 21 79104 Freiburg Germany; ^4^ Chemie, Arbeitskreis Buntkowsky Technische Universität Darmstadt Alarich-Weiss-Str. 8 64287 Darmstadt Germany; ^5^ Institut für Anorganische und Analytische Chemie Albert-Ludwigs-Universität Freiburg Albertstr. 21 79104 Freiburg Germany

**Keywords:** hydrogen, hyperpolarization, NMR spectroscopy, nuclear PHIP, *para*-to-*ortho* conversion

## Abstract

Molecular hydrogen has unique nuclear spin properties. Its nuclear spin isomer, *para*hydrogen (*p*H_2_), was instrumental in the early days of quantum mechanics and allows to boost the NMR signal by several orders of magnitude. *p*H_2‐_induced polarization (PHIP) is based on the survival of *p*H_2_ spin order in solution, yet its lifetime has not been investigated in aqueous or biological media required for in vivo applications. Herein, we report longitudinal relaxation times (*T*_1_) and lifetimes of *p*H_2_ ($\tau_{POC}$ ) in methanol and water, with or without O_2_, NaCl, rhodium‐catalyst or human blood. Furthermore, we present a relaxation model that uses *T*_1_ and $\tau_{POC}$ for more precise theoretical predictions of the H_2_ spin state in PHIP experiments. All measured *T*_1_ values were in the range of 1.4–2 s and $\tau_{POC}$ values were of the order of 10–300 minutes. These relatively long lifetimes hold great promise for emerging in vivo implementations and applications of PHIP.

## Graphical abstract

**Spin relaxation**: Longitudinal relaxation and *para*‐to‐*ortho* conversion of H_2_ are investigated in aqueous solutions and human whole blood. A relaxation model is proposed to simulate relaxation of the spin state of H_2_. *Para‐*H_2_ is a powerful source to enhance the signal of magnetic resonance, particularly promising for metabolic imaging and continuous hyperpolarization.

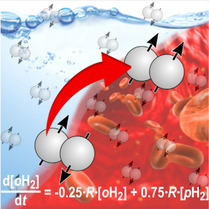

Atomic (H) and molecular hydrogen (H_2_) are two of the most fundamental and most frequently studied systems in chemistry and quantum mechanics. H_2_ has four nuclear spin eigenstates: three triplet states, referred to as *ortho*hydrogen (*o*H_2_), and one singlet state, referred to as *para*hydrogen (*p*H_2_). Its interesting spin properties have served to validate quantum mechanics some 100 years ago.[^1^, ^2^] More recently, it has become of great interest in the context of magnetic resonance imaging (MRI) and spectroscopy (NMR/MRS).

*p*H_2_ induced hyperpolarization (PHIP) or *p*H_2_ and synthesis allows dramatically enhanced nuclear alignment (PASADENA)[^3^, ^4^, ^5^] is an established method for nuclear spin hyperpolarization (HP) that has demonstrated signal enhancement by several orders of magnitude.[^6^, ^7^, ^8^, ^9^] One promising application of HP is to monitor the fate of hyperpolarized molecules non‐invasively, in vivo and real time.[^10^, ^11^, ^12^] Such HP tracers provide unique insights into biological mechanisms, e. g., tumor tissue metabolism. While dissolution dynamic nuclear polarization (dDNP),^[13]^ another HP method, is much further developed, established, and has been used in humans,[^11^, ^12^] PHIP methods are faster, less complex, and less costly.[^7^, ^14^, ^15^, ^16^, ^17^] PHIP is unique in generating HP in the liquid state and its continuous renewal.^[50]^

The longitudinal relaxation times, *T*_1_, of H_2_ nuclear spins have been investigated in gaseous phase,[^18^, ^19^] dissolved in organic solutions,[^20^, ^21^, ^22^, ^23^] or water.^[24]^ However, PHIP exploits the singlet spin order of *p*H_2_‐enriched H_2_ gas and hence the rate of *para*‐to‐*ortho* conversion (POC) – the lifetime of the singlet state, $\tau_{POC}$ – is much more relevant than *T*_1_.

It was found that $\tau_{POC}$ of gaseous H_2_ strongly depends on the surface of the container and ambient oxygen residuals.[^25^, ^26^, ^27^] In solvents, the POC depends on temperature and the presence of paramagnetic substances.[^20^, ^22^, ^28^, ^29^, ^30^, ^31^, ^32^, ^33^, ^34^] However, the lifetime of *p*H_2_ in the most relevant media for biomedical applications, (protio‐) water and biological fluids, has not been investigated yet. This shortcoming may be attributed to the difficulties that arise from i) detecting MR‐silent *p*H_2_ (total nuclear spin is 0) and ii) the overlapping resonances of H_2_ (chemical shift *δ*≈4.5 ppm) and H_2_O (*δ*≈4.7 ppm). At the same time, the POC rate, *R*_POC_=1/$\tau_{POC}$ , in these media is paramount: the slower the POC, the higher the yield of PHIP.

Here, we report *T*_1_ and $\tau_{POC}$ of H_2_ in various aqueous and biological media including solutions containing a water‐soluble rhodium‐catalyst and human blood.^[35]^

For our measurements, the samples were prepared in 5 mm NMR tubes. Note that these tubes were almost completely filled with solution and only a tiny gas phase remained (≈50 μL). The gas in the tube was replaced by pressurized H_2_ or *p*H_2_ and the tubes were sealed under pressure (7–10 bar). Next, the tube was turned upside down a few times to move the gas bubble through the solution to dissolve the hydrogen. Note that the dissolution kinetics of H_2_ are one to two orders of magnitude faster than the POC investigated here.^[36]^ Hence, when the NMR measurements were started (2–3 min after sealing the tubes), the dissolution was expected to be completed. ^1^H NMR spectra were acquired with a high‐resolution NMR spectrometer at 600 MHz and 300 K. POC was observed by measuring the growing *o*H_2_ MR signal. A more detailed experimental section is provided in the SI.

Measured *T*_1_’s were in the range of ≈1.4 to 2 s (Table 1). In water‐diluted samples of human whole blood, *T*_1_ decreased from (1.85±0.03) s for pure deionized (di) water, (1.7±0.1) s for 5 % blood to (1.30±0.03) s for 25 % blood. Hence, a *T*_1_ value of 0.69 s with a 90 % prediction interval of [0.64, 0.74] s is expected for pure human whole blood (first‐order rate assumed; value extrapolated from unweighted linear regression). A similar *T*_1_ value of (2.04±0.08) s for H_2_ in 99.75 % D_2_O at 303 K and 400 MHz was reported before.^[24]^

**Table 1:** Measured *T*_1_ values and POC times of H_2_ at 300 K and 600 MHz in methanol, water, deionized water (di‐H_2_O), saline solution and blood/water mixtures.

Solvent	*T*_1_ [s]	$\tau_{POC}$ [min]
methanol‐d_1_, 99 %		24.0±0.5
methanol‐d_4_, 99 %	1.482±0.002	23.7±0.2
methanol‐d_4_ (degassed)	1.503±0.002	293±2
di‐H_2_O	1.85±0.03	143±3
D_2_O, 99 %		160±6
di‐H_2_O (degassed)		192±2
saline solution	1.97±0.05	117±8
		
blood samples (D_2_O/H_2_O/blood fraction)		
75/25/0 %		156±5^[a]^
75/20/5 %		81±1
75/20/5 %	1.7±0.1	131±6
75/15/10 %		140±3
75/10/15 %		142±3
75/5/20 %		58±2
75/0/25 %	1.30±0.03	67±1
pure blood (extrapolated) [with 90 % prediction bounds]:	0.69 [0.64, 0.74]	25 [14, 86]

[a] The value for 75 % D_2_O, 25 % H_2_O was interpolated linearly from the measured values in pure samples.

In degassed methanol‐d_4_, where ambient O_2_ was replaced by N_2_, $\tau_{POC}$ =(293±2) min was obtained, which is consistent with a previous report [(320±60) min at 293.15 K; 200 MHz].^[20]^ In non‐degassed methanol‐d_4_ exposed to air, however, POC was significantly faster: $\tau_{POC}$ =(23.7±0.2) min.

In aqueous samples, the separation of the weak *o*H_2_ signal from the much larger (>10^4^‐fold) signal of H_2_O was more challenging. A 5^th^‐order polynomial function was fitted to the water resonance and subtracted to reveal the H_2_ resonance (Figure S1). In non‐degassed H_2_O, D_2_O and saline solution, POC times of (143±3) min, (160±6) min and (117±8) min were measured, respectively (see Figure S5). In degassed D_2_O, POC was even slower: $\tau_{POC}$ =(192±2) min (Table 1).

The ambient O_2_ significantly accelerates *R*_POC_ due to its paramagnetic properties.[^27^, ^30^] The degassing effect is stronger in methanol than in water, likely because O_2_ dissolves about 20 times better in methanol than in water.^[37]^

These results are very encouraging: because the POC in aqueous media is slow, only little polarization is lost on the time scales of PHIP experiments (seconds). For example, less than ≈7 % loss of *p*H_2_ are expected 10 min after dissolution in non‐degassed H_2_O.

The effect of blood on nuclear spin relaxation properties (e. g. *T*_1_) was described before,^[38]^ but its effect on *R*_POC_ was not investigated. To fill this gap, we measured $\tau_{POC}$ of *p*H_2_ in fresh, venous human whole blood. The ^1^H‐NMR resonance of H_2_ and $\tau_{POC}$ were successfully observed in water‐diluted blood samples up to a blood fraction of 25 % (Figure S6 and S7). For more than 25 %, the spectral lines became too broad for accurate quantification. In samples with 5 %, 10 % and 15 % blood in water, $\tau_{POC}$ was similar to the one measured in pure water; for a second sample with 5 % and samples with 20 % and 25 % blood, POC was shorter (Figure 1, Table 1). This variation may be explained by slight differences in sample preparation, oxygen‐saturation levels, fraction of deoxy‐hemoglobin^[38]^ and ethylenediaminetetraacetatic acid (EDTA) concentration,^[39]^ although none of these were evident. Also blood coagulation or separation may have been an issue, but was not apparent in the samples. Note that EDTA was used to prohibit coagulation.

**Figure 1:**
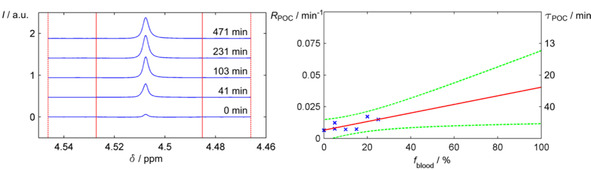
POC in human blood. Processed ^1^H NMR spectra of *o*H_2_ in 25 % blood in D_2_O (left) and seven POC rates *R*_POC_ measured in aqueous samples containing 0–25 % blood (right, crosses). *R*_POC_ in a pure blood was extrapolated from this data by an unweighted linear regression (solid line, 90 % prediction bounds as dashed lines; R^2^=0.49). NMR signal intensity, *I*, and blood fraction, *f*_blood_.

An unweighted linear regression of the reciprocal data suggested a $\tau_{POC}$ of ≈25 min in pure blood, with a 90 % prediction interval of [14, 86] min. Potentially, other methods may allow measuring POC in pure blood, e. g. Raman spectroscopy.[^40^, ^41^] However, the constituents of the blood may complicate the analysis of the *para* and *ortho* fractions as the concentration of H_2_ is relatively low.

To verify the long lifetime in blood, *p*H_2_ was dissolved in di‐H_2_O containing 10 % blood and PHIP was performed after 3, 13 and 33 min: all samples yielded similar ^13^C hyperpolarization. This result supports that *p*H_2_ barely decays during this time (see Figure 2).

**Figure 2:**
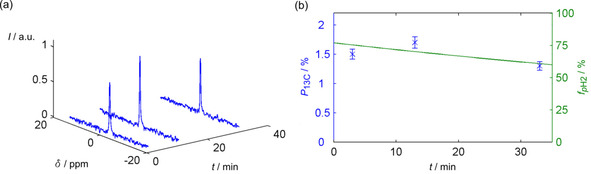
*p*H_2_ survives in human whole blood and allows ^13^C‐hyperpolarization. a) Hyperpolarized ^13^C NMR spectra and b) corresponding ^13^C polarizations (blue crosses) of hydroxyethyl‐1‐^13^C‐propionate‐d_3_ acquired at different times, *t*, after dissolution of *p*H_2_ in 10 % blood samples. In (b), the fitted *p*H_2_‐fraction, *f*_*p*H2_, measured in a sample with 10 % blood previously (Figure S7) was plotted for comparison (green line). Over 33 min after *p*H_2_ was dissolved, similar values of ^13^C polarization, *P*_13C_, were achieved by means of PHIP under PASADENA conditions.^[3]^ The PH‐INEPT+^[43]^ sequence was applied to hyperpolarize ^13^C nuclei. Thus, the *p*H_2_ fractions contained by the samples were of a similar order, which supports the long *p*H_2_ lifetime observed in blood. Intensity, *I*, and ^13^C chemical shift, *δ*. See the Supporting Information for more experimental details.

It is interesting to note that H_2_ gas is considered as a promising therapeutic, antioxidant gas for many applications.^[42]^ H_2_ rapidly diffuses into tissues and cells, has either no or very little side effects and proceeds fast into the circulatory system after breathing.^[42]^

Here, venous blood was used, which has a higher content of deoxy‐hemoglobin, a prominent source of relaxation,^[38]^ than arterial blood. Thus, a $\tau_{POC}$ >10 min of H_2_ in vivo appears not unlikely and it may be feasible to accumulate *p*H_2_ in vivo.

In addition to *p*H_2_, PHIP experiments require a target substrate and a hydrogenation catalyst. The substrates are usually small organic molecules that do not interact with H_2_ and won't cause significant relaxation. In contrast, the catalysts are typically metal‐organic complexes that are involved in exchange reactions with H_2_ and thus may result in significant loss of the effective *p*H_2_ lifetime in solution.

When a rhodium‐based water‐soluble PHIP catalyst (see SI) was added to D_2_O, the dynamics of *p*H_2_ were remarkably different. Note that prior to these experiments, the catalyst stock solution was kept for 90 min at a 1 bar H_2_ atmosphere to remove the protective norbornadiene.^[35]^ Two effects were observed, which depended on the concentration of the catalyst [Figure 3, Eq. (S2)]: (a) an increase of the *o*H_2_ MR signal, likely attributable to POC, followed by (b) a decrease of *o*H_2_ signal. In samples with *c*_cat_=1 mM and 2 mM, the *o*H_2_ resonance vanished after 100 min or 50 min, respectively. At the same time, HD signal emerged and vanished (*δ*=4.48 with *J*_HD_ coupling≈45 Hz).

This observation indicated a catalyzed isotope exchange (IE) of H_2_ with the solvent: H_2_→DH→D_2_, along with D_2_O→HDO. Note, that IE of hydrogen in the presence of other transition‐ and alkali‐metal complexes has been discussed previously[^29^, ^33^, ^44^] and is not the main subject of this work.

A model that considers POC and IE was derived and fitted to the data, assuming a constant IE rate, *R*_IE_ [Eq. (S4)]. However, *R*_IE_ seemed to increase over time in presence of H_2_ (Figure 3). Because of this simplification, the derived model did not perfectly fit the data, thus resulted in an underestimation of $\tau_{POC}$ and a time‐averaged value of $\tau_{IE}$ (Table 2). The observed POC and IE rates depended approximately linearly on the concentration of the catalyst.

**Figure 3:**
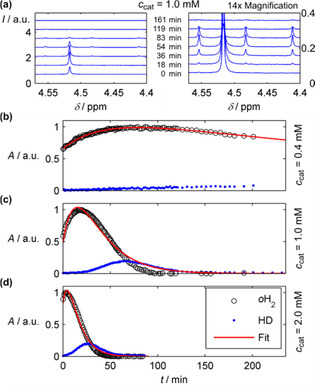
Dynamics of *p*H_2_ in aqueous solutions with catalyst of different molarity. a) ^1^H‐NMR spectra and b–d) time‐evolution of the integrals over the *o*H_2_ (black circles) and HD signals (blue dots) and fitted function (red line) in samples with 0.4 mM (b), 1.0 mM (c) and 2.0 mM (d) catalyst in D_2_O, respectively. (a) and (c) correspond to the same data set. Intensity, *I*, and integrated peak area, *A*, of the NMR signal.

**Table 2:** Measured POC and IE times,$\;\tau_{POC}$ and$\;\tau_{IE}$ , of H_2_ in D_2_O with different molarity of Rh‐catalyst.

*c*_cat_ in D_2_O [mol L^−1^]	*τ*_POC_ [min]	*τ*_IE_ [min]
0.0	160±6	–
0.4	45.4±2.7	510±30
1.0	33.6±1.0	25.9±0.4
2.0	14.0±0.4	10.7±0.2

To investigate the increase of *R*_IE_, the hydrogenation rate of the substrate was measured by NMR after the same catalyst solution was in contact with *p*H_2_ at ≈3 bar for 5 min or 1 h (see SI for more details).

The catalyst activity was found to be better after 60 min exposure, which rules out a deactivation of the catalyst under H_2_ exposure (Figure S8). However, PHIP requires pair‐wise hydrogenation of *p*H_2_ and conservation of the spin order during this reaction. Hence, the observation does not necessarily mean that the H_2_‐exposed form of the catalyst is favorable for PHIP experiments. This issue deserves further investigation.

It should be noted that the lifetimes in presence of the water‐soluble Rh‐catalyst reported here were much longer than in a previous study. In that study, another Rh‐based catalyst in acetone yielded a much shorter POC: fast singlet‐triplet conversion of H_2_ at the catalyst (intermediate) resulted in a partial negative line (PNL) effect.^[34]^ Apparently, with the water‐soluble catalyst complex used here, either the exchange rates are much lower or the intermediate complexes do not form. PNLs were not observed here, which could be a great advantage over the non‐water‐soluble ligand and may help designing more effective PHIP catalysts in future.

Also when the water‐soluble catalyst is present, it appears that POC and IE will play a minor role with respect to achieved polarization yields in PHIP experiments as the hydrogenation time is typically short (<10 s). For instance, in 2 mM catalyst solution, no more than ≈3 % *p*H_2_ are lost within 10 s (assuming the time constants measured here).

*T*_1_ relaxation of H_2_ in solution is expected to be governed largely by dipole–dipole (DD) interactions, with some contributions from spin‐rotation (SR) (see the Supporting Information for details).^[45]^ While *p*H_2_ is immune to intramolecular DD and SR relaxation, intermolecular DD interactions are the main source for relaxation in solutions,^[46]^ although it is challenging to predict the impact ab initio.

Here, we propose to combine an intramolecular dipole–dipole (DD) relaxation superoperator, ${\hat{\hat{R}}}^{DD}$ , as the main source of H_2_ *T*_1_‐relaxation,^[45]^ and another relaxation superoperator based on the model of uncorrelated local fluctuating fields (LFF),^[47]^ ${\hat{\hat{R}}}^{LFF}$ , to simulate both *T*_1_ relaxation and POC [Eq. (1)]:

(1) $${\hat{\hat{R}}}^{H_{2}}\left[T_{1},\tau_{POC}\right]={\hat{\hat{R}}}^{DD}\left[0.74\;\;A,0.1922\;\;ps\right]\cdot \left(\frac{1}{T_{1}}-\frac{1}{2\tau_{POC}}\right)\cdot 1\;\;s+{\hat{\hat{R}}}^{LFF}\left[2\tau_{POC}\right]$$

We find that the superposition of these two relaxation superoperators can be used to describe the relaxation of H_2_ with any given values of *T*_1_ and $\tau_{POC}$ (*T*_1_ ≪ $\tau_{POC}$ ). This phenomenological relaxation superoperator will be very useful for quantitative simulations of polarization yield in PHIP experiments, because it allows simple and more accurate prediction of the spin state of H_2_[^48^, ^49^] (see the Supporting Information for more details and an example).

To conclude, we measured POC in aqueous solutions, whole blood–water mixtures and aqueous solutions with a water‐soluble Rh‐catalyst. All values were >10 min and degassing significantly extended POC up to 200 min. Degassing had a stronger effect in methanol than in water, likely as O_2_ dissolves about 20‐fold better in methanol. Since the duration of a PHIP experiment is usually of the order of seconds, POC appears to have little impact on the polarization. The long POC in blood suggest that an accumulation of *p*H_2_ in vivo may be feasible. A relaxation model was proposed and used to simulate the relaxation of the spin state of H_2_ more accurately. The model exploits phenomenological properties (measured *T*_1_ and $\tau_{POC}$ values) to predict the actual spin state of H_2_, allowing a more quantitative simulation of PHIP.

## Experimental Section

The experimental section, additional experimental results, details and an example of theory application are provided in the Supporting Information.

## Conflict of interest

The authors declare no conflict of interest.

## Supporting information

As a service to our authors and readers, this journal provides supporting information supplied by the authors. Such materials are peer reviewed and may be re‐organized for online delivery, but are not copy‐edited or typeset. Technical support issues arising from supporting information (other than missing files) should be addressed to the authors.

Supplementary
