# Supplementary material for: Lifetime of Parahydrogen in Aqueous Solutions and Human Blood
Source: Chemphyschem. 2019 Sep 12;20(19):2408–12. doi: 10.1002/cphc.201900670 (PMC7687157; doi:10.1002/cphc.201900670)
Supplement: Supplementary file 1 — Supplementary [file CPHC-20-2408-s001.pdf]

CHEMPHYSCHEM

Supporting Information

© Copyright Wiley-VCH Verlag GmbH & Co. KGaA, 69451 Weinheim, 2020

Lifetime of *Para*hydrogen in Aqueous Solutions and Human Blood

Andreas B. Schmidt,* Jakob Wörner, Andrey Pravdivtsev, Stephan Knecht, Harald Scherer, Stefan Weber, Jürgen Hennig, Dominik von Elverfeldt, and Jan-Bernd Hövener*© 2019 The Authors. Published by Wiley-VCH Verlag GmbH & Co. KGaA. This is an open access article under the terms of the Creative Commons Attribution License, which permits use, distribution and reproduction in any medium, provided the original work is properly cited.

Table of Contents

I. METHODS 2

Setup and preparation of samples 2

Data acquisition 3

II. Measurement of T_1 relaxation times 3

III. Measurement of thermally equilibrated H_2 and gas leakage 4

IV. Effect of the residual gas phase on the observed POC time 5

V. Measurement of POC times 6

VI. Measurement POC in the presence of isotope exchange of H_2 with the solvent 8

VII. PHIP experiments in human blood 9

VIII. Measurement of the catalyst’s activity 10

IX. Phenomenological relaxation superoperator of H_2 in liquids 10

I. METHODS

Setup and preparation of samples

Parahydrogen: The pH_2 fraction of normal H_2 gas (purity of >99.999%) was enriched to ≈90 % as was described previously.[1] pH_2-enriched gas was filled into an aluminum gas cylinder (0.5 L, AL 0,5 LTR BG DIN477-1 NR. 1, gasetechnik24, Germany) and was used on demand.

NMR setup: NMR signals were detected using a 600-MHz NMR spectrometer (Topspin 3.2; Avance III HD 600 MHz, Bruker, Germany) in combination with a 5 mm probe (5-mm-PABBO-BB/^{19}F-^{1}H/D-Z-GRD or 5-mm-PATXI-^{1}H/D-^{13}C/^{15}N-Z-GRD, Bruker, Germany).

Sample preparation: Investigated solvents were filled into 5 mm NMR pressure tubes (5 mm quick pressure valve tube, Wilmad-LabGlass, USA). Unless stated differently the tubes were filled with solution until only a tiny gas bubble remained in the tube (≈50 µL). The gas was replaced by a pressurized H_2 atmosphere and the NMR tube was sealed under pressure (7–10 bar). The tiny bubble was then guided through the sample several times with the help of a vortexer (Lab dancer, IKA, Germany). The sample was prepared and placed in the NMR system approximately one to two minutes after H_2 was added to the tube. Note, that NMR acquisitions started after 2 – 3 minutes after H_2 insertion to achieve equilibration of temperature and convection of the sample.

Degassing: Oxygen was replaced by N_2 using Schlenk technique and three subsequent freeze-pump-thaw cycles (vacuum pump BZ43, Pfeiffer, Germany).

Saline solution: Anhydrous sodium chloride was dissolved in deionized water with a molarity of 154 mM (9 g/L, ≥99 % NaCl, Sigma-Aldrich, USA).

Catalyst solution: A commonly used water-soluble PHIP catalyst was formed by mixing 22 mM of bis-phosphine ligand (1,4-bis-[(phenyl-3-propane sulfonate) phosphine] butane disodium salt, MW = 562.53 g/mol, MDL number MFCD15144866, Sigma Aldrich, MO, USA) and 20 mM of rhodium complex (bis(norbornadiene)rhodium (I) tetrafluoroborate, MW = 373.99 g/mol, CAS 36620-11-8, StremChemicals, MA, USA) in D_2O.[2–4] The resulting catalyst solution was then kept under H_2 atmosphere at ambient pressure and room temperature for 90 min to remove the remaining norbornadiene from the complex.[5] Note that this step usually takes place during the PHIP experiments but was done here prior to the experiments to avoid this reaction with H_2 during a measurement. After degassing the sample, NMR tubes were filled with some catalyst solution from this batch and mixed with different amounts of D_2O yielding catalyst solutions of different molarities (0.4–2 mM).

Human blood: In pure blood the H_2 resonance was concealed by the water signal. Likely line broadening was caused by exchange reactions of H_2O with some of the other present molecules and, in addition, the static magnetic field was poorly shimmed as paramagnetic blood constituents were present and NMR tubes were fully filled. Hence, six different samples of blood were diluted with different amounts of water, yielding samples with 5 % to 25 % of blood and the POC time in pure blood was interpolated from the acquired data (125 – 625 µL blood and 1,800 µL D_2O were added to an NMR tube; then the tube was filled with 0–500 µL H_2O; total volume was ≈2.5 mL). Venous blood was taken from one of the experimenters (ABS) with his permission and by himself using syringes containing the anticoagulant ethylenediaminetetraacetic acid (EDTA) (2.7 mL, EDTA K S-Monovette, Sarstedt AG & Co. KG, Germany) and a 21-gauge needle with a butterfly clip (Safety-Multifly-Needle, Sarstedt AG & Co. KG, Germany). Blood was taken freshly before every experiment. Note that also the NMR tube and pipettes were coated with EDTA before filling the blood (MW = 292.25 g/mol, CAS 60-00-4, AppliChem GmbH, Germany).

Data acquisition

Adjustments: Before pH_2 was added to the samples filled with different solutions, they were placed in the NMR magnet to perform adjustments. In experiments where a long POC was expected (> 10 min; all samples but blood, non-deoxygenated methanol and catalyst solution) another automated adjustment was performed when the pH_2 containing sample was placed in the NMR magnet.

Longitudinal relaxation times: T_1 of dissolved H_2 was measured using H_2 gas at thermal equilibrium using the inversion recovery method (pulse sequence: 180°-Δt-90°, with different recovery times Δt; 4 averages). The Topspin pulse sequences "t1ir" and "t1ir1d" were used.

***Para*-to-*ortho* conversion:** The kinetics of *para*-enriched H_2 gas dissolved in liquid samples was investigated at 300 K by ^{1}H-NMR. Samples were monitored over a period of 1-10 hours, depending on the (expected) speed of the POC (30° pulse, 8 or 16 averages, acquisition time of ≈3 s, repetition time of each average of T_R: ≈4 s (≥ $2 \cdot T_1$,→ more than 93 % of the signal recovered)); first data was typically acquired 2-3 min after pH_2 insertion. Note that the sample was scanned more frequently at the beginning of the POC (where changes were expected to be larger) than at the end of the conversion.

As the H_2 resonance is small compared to adjacent H_2O signals, experiments were undertaken at a high magnetic field of 600 MHz (14.1 Tesla) to separate the NMR lines. However, as the H_2O resonances were broad and much larger than the H_2 signals, the latter still was overlain by the slope of the water signal in aqueous samples. The H_2 signal was isolated by fitting polynomial functions to the H_2O slope left and right of the H_2 resonance and subtracting it from the data (Fig. S1). Note that this procedure was not possible in pure whole blood, where the H_2 resonance could not be identified under the broadened H_2O peak. Hence, the POC in pure blood was extrapolated from POC data acquired in diluted blood samples to reduce the signal and linewidth of H_2O.

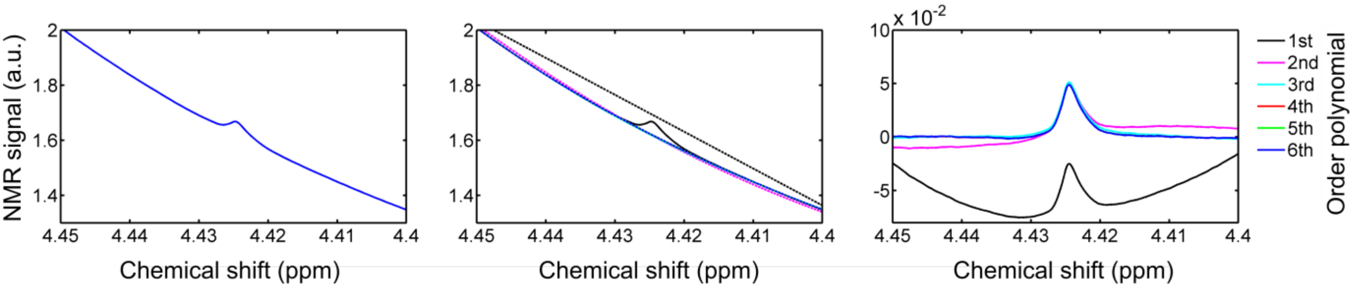

Figure S1. Evaluation of H_2 in H_2O. Direct integration of the H_2 resonance on the flank of the water signal (left) was not possible as B_0-field drifts (experiment times of several hours) and changes of the shim and baseline interfered. Instead the H_2 resonance was isolated by fitting polynomials to the water flank in a small interval to the left and right of the H_2 peak (middle). Different polynomial orders were tested and subtracted from the spectral data (right). Qualitatively, 5th order polynomials were sufficient to evaluate H_2 in aqueous solutions and were used in this work.

II. Measurement of T_1 relaxation times

Data acquired by the "t1ir1d" method were evaluated using a custom-written script (Matlab, MathWorks, USA). For data acquired with the Topspin "t1ir" routine the T_1 was obtained from the pseudo-2D data using the Topspin 3.2 integrated relaxation analysis module (see Fig. S2).

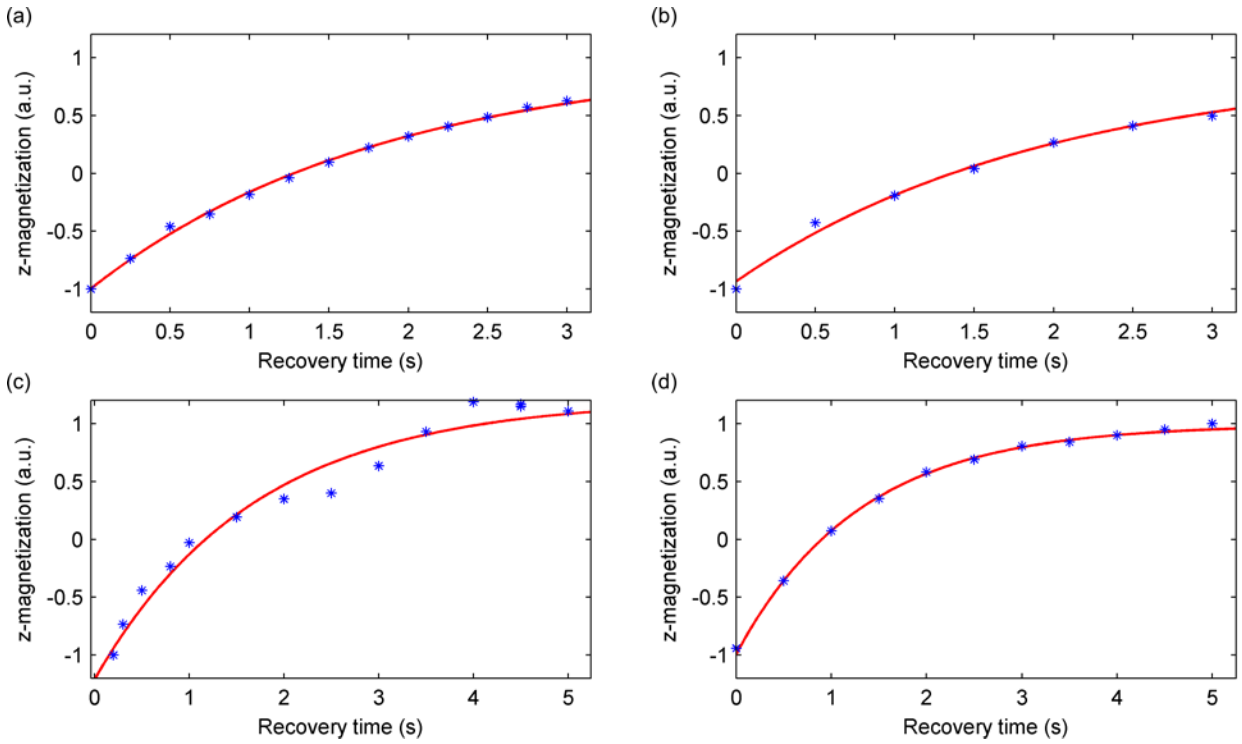

Figure S2. Longitudinal relaxation of H_2 in water, saline solution and human blood. T_1 values of dissolved H_2 were measured in samples of di-H_2O (a) saline solution with 9 g/L NaCl in di-H_2O and water-blood mixtures with 5 % blood (c) and 25 % blood (d) by ^{1}H-NMR using the inversion-recovery method. An exponential saturation curve (solid red line) was fitted to the measured data (blue asterisks) and longitudinal relaxation times were extracted. T_1 values of (1.85 ± 0.03) s, (1.97 ± 0.05) s, (1.7 ± 0.1) s and (1.30 ± 0.03) s were determined for H_2 in (a), (b), (c) and (d), respectively. For a pure blood sample a T_1 of 0.69 s with 90 % prediction interval of [0.64, 0.74] s was extrapolated by linear regression of the reciprocal T_1 values. Note that D_2O was added to the samples to lock the magnetic field and reduce the water signal (10 % in (a), 15 % in (b) and 75 % in (c) and (d)).

III. Measurement of thermally equilibrated H_2 and gas leakage

To measure how good the NMR tubes were sealed thermally equilibrated H_2 gas at room temperature (rtH_2) was dissolved in methanol-d_1 (99 %) at 7 bar and was monitored over a period of 3 hours. A slow, approximately linear decrease of the oH$_2$ MR signal with a slope of -(2.9 ± 1.4) % / hour was observed. In contrast, the signals of methanol remained constant over time within one σ (Fig. S3). We attribute the decaying H_2 MR signal to leaking of H_2 from the NMR tube. Since this effect is small, it was ignored for most evaluations. Only for degassed water and methanol, which were monitored for longer periods of ≈10 h, gas leaking was considered by adding a linear term to the fitted function, Eq. (S1) (see Fig. S5 and S7).

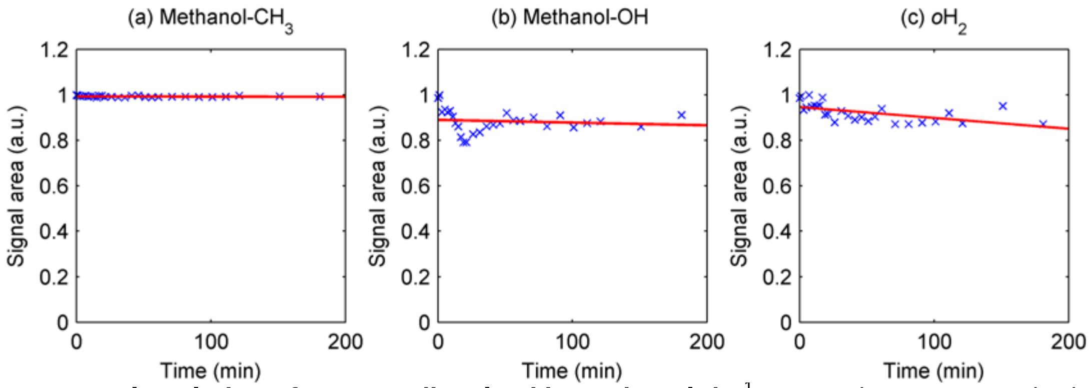

Figure S3. Temporal evolution of rtH_2 gas dissolved in methanol-d_1. ^{1}H-NMR data were acquired over a period of 180 min after rtH_2 gas was dissolved in methanol-d_1. An unweighted linear regression (dashed line) revealed that the signal of H_2 (circles; 7 bar, 300 K, H_2 bubble ≈50 µL) decreased by -(2.9 ± 1.4) % / hour, likely due to H_2 gas leaving the NMR tube. The ^{1}H-NMR signals of methanol-CH_3 (blue asterisks) and methanol-OH (data not shown) remained constant within one σ of the fit (CH_3: -(0.06 ± 0.08) %/hour; OH: -(1 ± 1) %/hour). Note that a prominent decrease of the H_2 MR signal was also observed in other samples that were monitored over long periods of time (see Fig. S5 and Fig. S7).

IV. Effect of the residual gas phase on the observed POC time

We found that the residual gas phase in the NMR tube had an impact on the results. When the volume of the residual gas (V_{gas}) in a 5 mm tube filled with methanol (10:1 methanol : methanol-d_1) was reduced from ≈ 1.6 mL to ≈ 40 µL in eight steps, τ_{POC} dropped linearly from (80 ± 20) min to (36 ± 7) min. An unweighted linear regression of the data suggested a τ_{POC} of (36 ± 5) min for V_{gas} = 0, which is similar to the values measured for V_{gas} = 40 µL and 250 µL within one standard error (Fig. S4). Essentially, this effect prolongs the measured POC in solution, because τ_{POC} of gaseous pH_2 is much longer than τ_{POC} of dissolved pH_2.[18,24] Within the experimental error, however, the effect was negligible for volumes of 50 µl or less, which were used in all following experiments.

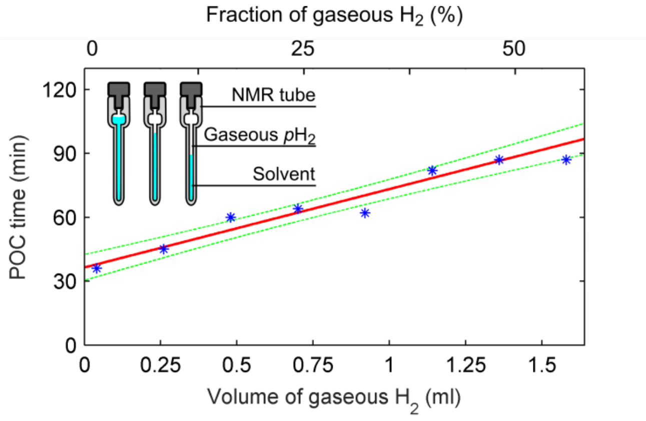

Figure S4. POC time, τ_{POC}, as function of gas phase volume, V_{gas}, and fraction, f_{gas}, in the NMR tube. As V_{gas} was reduced, the measured τ_{POC} times (blue asterisks) were shortened linearly (linear fit: solid red line, with 95 % confidence interval in dashed green).

V. Measurement of POC times

FID raw data was evaluated using custom-written software (Matlab); some functions were provided by Dr. Peter Blümler, Mainz (personal communication).

1. The FID was zero-filled 4 times before Fourier transformation and phase correction was applied. A 1-Hz exponential line broadening was applied to reduce noise.
2. For non-deuterated aqueous solutions, a 5th-order polynomial was fitted to the slope of the H_2O resonance that partially overlaid the H_2 resonance. The fitted function was than subtracted from the data to isolate the H_2 resonance.
3. Finally, the H_2 peak was integrated numerically.

Both, T_1 and the time constant of the POC, τ_{POC}, were extracted from a mono-exponential saturation function that was fitted to the corresponding data:

$$\mathrm{f}(t) = A - (A - B) \cdot \exp\left(-\frac{t}{T}\right) . \qquad \text{(S1)}$$

In these functions, *A* is the NMR signal of the sample at thermal equilibrium. *B* is the initial signal at time $t = 0$ (in case of T_1 $B = -A$; in case of POC $B \geq 0$, accounting for an initial oH_2 fraction of $[oH_2] \geq 0$).

Note, though, that the exact amount of dissolved H_2 is neither relevant for the quantification of T_1, nor for measurements of POC (if the process can be assumed to be in the pseudo 1st order kinetics regime, see below Section VI).

Below, NMR spectra and POC kinetics of H_2 in saline solution (Fig. S5 (a) and (b)), water (Fig. S5 (c) and (d)) and blood - water mixtures (Fig. S6 and S7) are shown.

We note that in contrast to a previous report[6], negative oH_2 NMR lines were not observed in the presence of O_2, neither in methanol or water, nor in water-diluted blood samples (see below). Partially, this may be attributed to the lower pH_2 enrichment used here (≈90 % before experiments and ≈80 % when the first spectra were acquired, as compared to the 100 % pH_2 in Ref. [6]). Additionally, the oxygen solubility in water and blood is low and O_2-catalyzed POC may be a secondary source of relaxation.

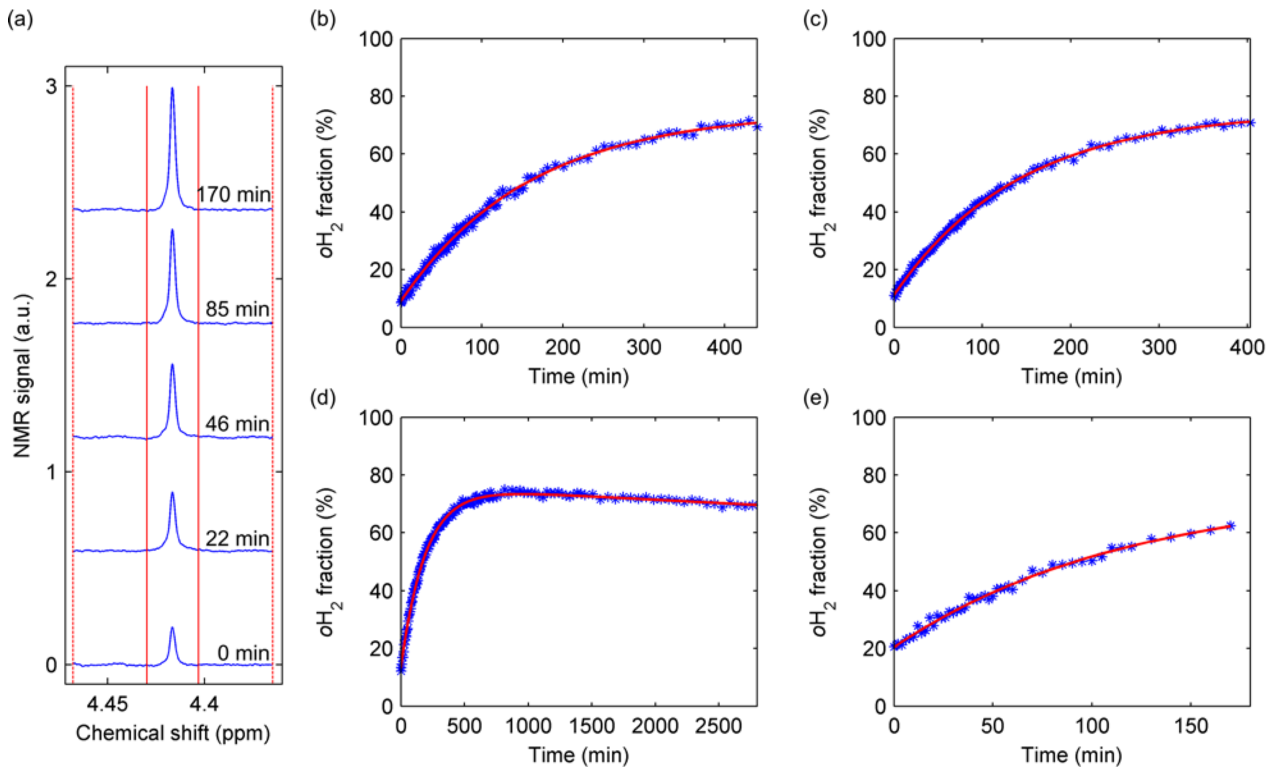

Figure S5. Increasing *ortho*-fraction of H_2 in water and saline solution over time. ^{1}H NMR signals of H_2 in saline solution acquired at different times during POC (a) and time evolution of the *o*H_2 fraction in di-H_2O (b), D_2O (c), degassed di-H_2O (d) and saline solution with 9 g/L NaCl in di-H_2O (e). Para-enriched H_2 gas was dissolved in the aqueous samples. The H_2 resonance gradually increased over time corresponding to an increasing *o*H_2-fraction (data represented by blue asterisks in (b)-(e)). Integration of the single spectra and plotting against time revealed the expected behaviour of the H_2 signal during POC. POC time constants of τ_{POC} = (143 ± 3) min, τ_{POC} = (160 ± 6) min, τ_{POC} = (192 ± 2) min and τ_{POC} = (117 ± 10) min were extracted from a fitted exponential saturation function (red solid line in (b)-(e)). In (d) a linear decrease of the H_2 signal was obvious after POC had finished, likely corresponding to H_2 leakage (see Section VI). This reduction was corrected by adding a linear term to the fit function, Eq. (S1). This term revealed a leakage of H_2 of -(0.136 ± 0.009) %/hour in sample (d).

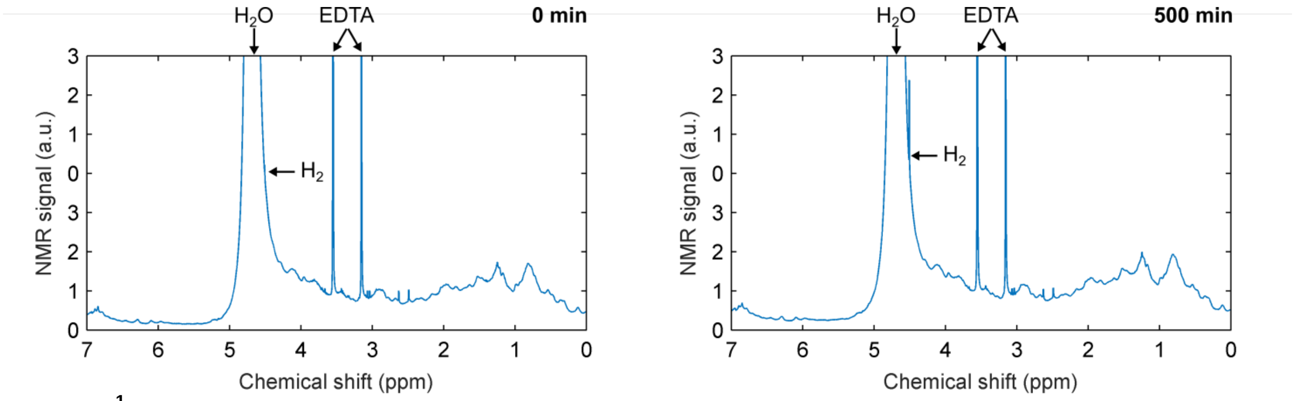

Figure S6. ^{1}H NMR of H_2 in water-diluted human blood. Venous human blood was taken from the crook of the arm using a butterfly cannula and an EDTA blood tube. An NMR pressure tube was filled with 75 % D_2O and 25 % of the blood sample and *p*H_2 gas was dissolved in it (≈7 bar). ^{1}H NMR spectra were acquired over 500 min and H_2O, H_2 and EDTA[7] resonances were identified. Other resonances were detected from blood constituents. The first (0 min; left) and last (500 min; right) NMR scan of the experiment are shown.

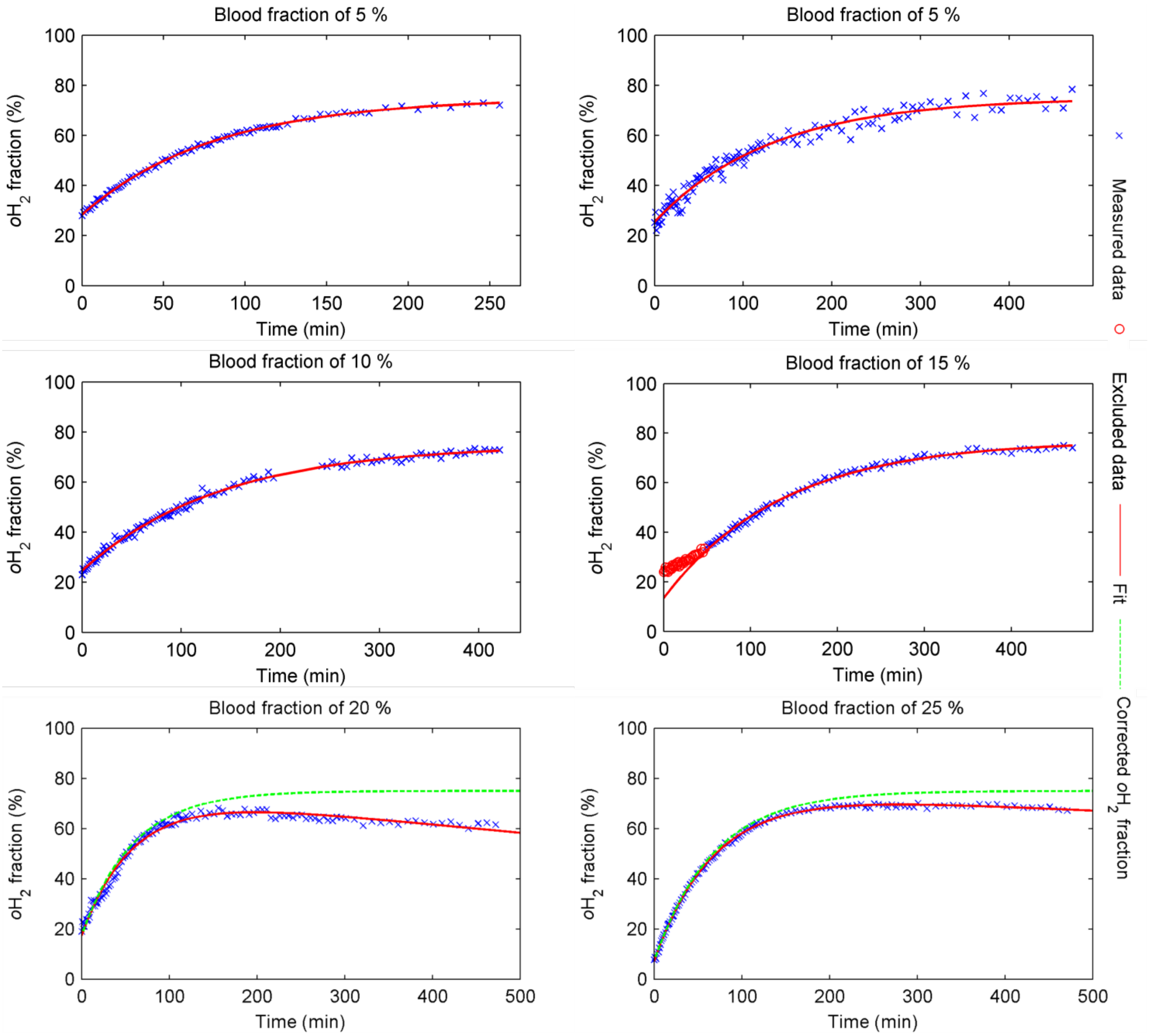

Figure S7. POC of H_2 gas in diluted blood samples with different blood fractions. In pure blood samples the H_2 resonance could not be separated from the broad H_2O resonance. Thus, NMR tubes were filled with 75 % D_2O and different amounts of human blood (5-25 %; two different samples with 5 % blood – water mixture were measured). The rest of the tube was filled with deionized H_2O to yield similar H_2O-D_2O ratios in the samples and only a small pressurized pH_2 bubble was enclosed in the tubes. Measured signal integrals of the H_2 resonance over time are represented by blue crosses. Mono-exponential curves were fitted to the data to determine τ_{POC} and are plotted in solid red lines.

Remarks:

- 10 % blood sample: From ≈200 – 230 min no data was acquired; additionally, a sudden change of phase in the signal was observed 84 min after onset and was corrected before evaluation.
- 15 % blood sample: Apparent POC was significantly slower at the beginning of the experiment, most likely due to errors of the baseline correction for a small H_2 resonance; this first data, which is indicated by red open circles in the figure, was ignored in the evaluation and a POC time similar to pure water was measured.
- 20 and 25 % blood samples: After POC had finished a decrease of the oH_2 signal, likely because of gas leakage, was visible. For evaluation a linear term was added to the fit function, Eq. (S1) and suggested a leakage of -(2.0 ± 0.2) %/hour and -(0.93 ± 0.08) %/hour in the samples with 20 % and 25 % blood, respectively. The corrected oH_2 fraction is shown in dashed green.

VI. Measurement POC in the presence of isotope exchange of H_2 with the solvent

For POC in the presence of a catalyst in D_2O, the following reactions were assumed to take place:

$$p\mathrm{H}_2 \underset{0.25\cdot R'_{\mathrm{POC}}}{\overset{0.75\cdot R'_{\mathrm{POC}}}{\rightleftarrows}} o\mathrm{H}_2;$$
$$\mathrm{H}_2 + \mathrm{D}_2\mathrm{O} \xrightarrow[R'_{\mathrm{IE}}]{} \mathrm{HD} + \mathrm{HDO}; \qquad \text{(S2)}$$
$$\mathrm{HD} + \mathrm{D}_2\mathrm{O} \xrightarrow[0.5\cdot R'_{\mathrm{IE}}]{} \mathrm{D}_2 + \mathrm{HDO};$$

with R'_k the rate constant of process 'k', namely 'POC' and isotopic exchange 'IE' (in case of the latter it was found that it is not constant (main article Fig. 3). The rates in the reaction $pH_2 \leftrightarrow oH_2$ reflect the amount of spin states of the two H_2 nuclear spin isomers (one state for *para*hydrogen and three states for *ortho*hydrogen).

The rate in the third reaction in Eq. (S2) considers the possibility of an exchange of deuterium atoms between HD and D_2O. For simplification, the concentration [D_2O] was assumed being constant and [D_2O] » [HDO] and [H_2O]. Thus reactions with HDO or H_2O involved can be neglected and, second order rates R'_{IE} (and equations) can be changed as follows: $R'_k \cdot [D_2O] = R_k$ (pseudo-first order regime).

The following rate equations were derived:

$$\begin{aligned}
\frac{\mathrm{d}[p\mathrm{H}_2]}{\mathrm{d}t} &= -0.75\cdot R_{\mathrm{POC}}\cdot[p\mathrm{H}_2] + 0.25\cdot R_{\mathrm{POC}}\cdot[o\mathrm{H}_2] - R_{\mathrm{IE}}\cdot[p\mathrm{H}_2];\\
\frac{\mathrm{d}[o\mathrm{H}_2]}{\mathrm{d}t} &= 0.75\cdot R_{\mathrm{POC}}\cdot[p\mathrm{H}_2] - 0.25\cdot R_{\mathrm{POC}}\cdot[o\mathrm{H}_2] - R_{\mathrm{IE}}\cdot[o\mathrm{H}_2];\\
\frac{\mathrm{d}[\mathrm{HD}]}{\mathrm{d}t} &= R_{\mathrm{IE}}\cdot[p\mathrm{H}_2] + R_{\mathrm{IE}}\cdot[o\mathrm{H}_2] - 0.5\cdot R_{\mathrm{IE}}\cdot[\mathrm{HD}];\\
\frac{\mathrm{d}[\mathrm{D}_2]}{\mathrm{d}t} &= 0.5\cdot R_{\mathrm{IE}}\cdot[\mathrm{HD}];
\end{aligned} \qquad \text{(S3)}$$

The kinetic time evolution of [oH_2] was obtained by solving these coupled ordinary differential equations (ODE's) analytically (initial conditions at time t_0: $[pH_2]_{t0} = 1$, $[oH_2]_{t0} = [HD]_{t0} = [D_2]_{t0} = 0$) using Mathematica 11, Wolfram Research, USA:

$$[o\mathrm{H}_2](t) \;=\; \exp\left(-R_{\mathrm{IE}}\cdot(t-t_0)\right)\cdot\left(A - A\cdot\exp\left(-R_{\mathrm{POC}}\cdot(t-t_0)\right)\right), \quad \text{(S4)}$$

with time delay t_0 considering the proceeded conversions at $t = 0$ and A adjusting the height of the curve. Conversion times, τ, were obtained by fitting this curve to measured data and by taking the reciprocal value of $R_i = \tau_i^{-1}$.

VII. PHIP experiments in human blood

Venous human whole blood was diluted with di-H_2O (1:7) and was kept in a round-bottom flask under pH_2 atmosphere for >10min. A magnetic stir bar was used to improve the uptake of pH_2 in solution. Three samples of 4 ml blood-water mixtures were filled into three syringes and were sealed using a plug. After different waiting times (3 min, 13 min and 33 min after filling) the pH_2-containing blood samples were injected into 1 ml catalyst-substrate solution held in a 5 ml Eppendorf tube kept in the isocenter of a 7 T preclinical MRI system (MRI system: BioSpec 70/20, Bruker, Germany). The substrate: hydroxyethyl-1-^{13}C, 2,3,3-$^{2}H_3$-acrylate (HEA), transformed to hydroxyethyl-1-^{13}C, 2,3,3-$^{2}H_3$-propionate (HEP) after hydrogenation. The final concentrations were 2 mM catalyst and 1 mM HEA in 10 % blood and 90 % water (5 ml total volume). The blood-water mixture was injected over 3 s; after another 5 s the PH-INEPT+ sequence[9] was applied to achieve ^{13}C hyperpolarization. A ^{1}H-^{13}C volume resonator was used for RF-excitation and receiving the ^{13}C-MR signal. After 3, 13 and 33 min,

^{13}C-HP of 1.5 %, 1.7 % and 1.3 % were measured, respectively. HP was quantified with respect to the ^{13}C signal of 5 mL acetone recorded in a separate measurement (50 averaged scans, sample at thermal equilibrium and with natural abundance ^{13}C of ≈1.1 %). Note that the ^{13}C signal of the samples at thermal equilibrium is several orders of magnitude too low to be detected by the MRI system. The error of a similar experiment was estimated to ± 10 %,[4] indicating that the measured values here were equal within one σ.

VIII. Measurement of the catalyst's activity

In order to investigate that the PHIP catalyst did not undergo deactivation during the storage under H_2 atmosphere prior to the experiments, we measured its hydrogenation activity. Two samples were prepared consisting of 1 mM catalyst in 600 µL methanol-d_4. One sample (Fig. S9 (a)) was bubbled with H_2 for 5 minutes (a) and the other was bubbled for 60 minutes (b) at 3 bar (Fig. S9 (b)). Subsequently, 440 mM of styrene were added as the substrate to the sample and the conversion of styrene to ethylbenzene was monitored by recording NMR spectra every 3 seconds at 300 K and constant H_2 3 bar $p$$H_2$. Under these conditions, the first-order hydrogenation process of styrene (S) to ethylbenzene (E) ($\frac{\mathrm{d}}{\mathrm{d}t}[\mathrm{E}](t) = k_{\mathrm{hyd}}[\mathrm{S}](t)[\mathrm{H_2}][\mathrm{Cat}]$) can be approximated by a zero order kinetics and we found rates of $k_{\mathrm{hyd}} = 2\ [\mathrm{s^{-1}mM^{-2}}]$ and $k_{hyd} = 10\ [\mathrm{s^{-1}mM^{-2}}]$ for the samples (a) and (b), respectively. Thus we conclude that the storage under H_2 does not lead to any meaningful deactivation during the storage times, but rather that H_2-exposal and removal of norbornadiene from the system increases the catalytic activity, which, however, deserves further investigations.

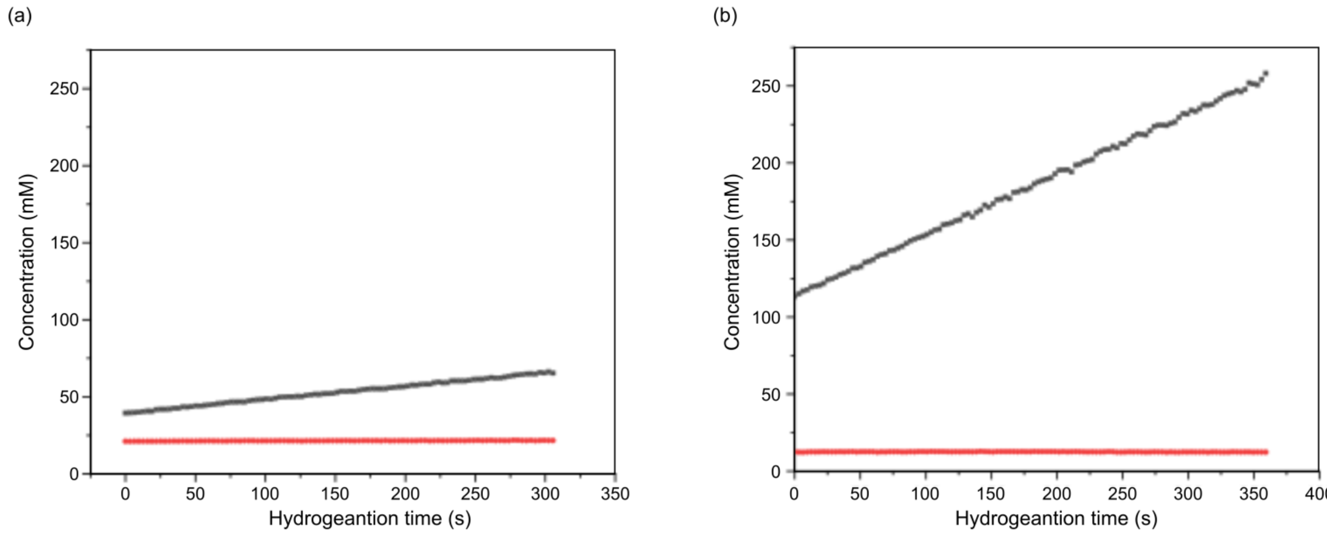

Figure S8. Measurements of the activity of the catalyst after exposure to H_2. The conversion of styrene to ethylbenzene was monitored (black dots) for two samples with 1 mM catalyst that were exposed to 3 bar H_2 for different times (5 min (a) and 60 min (b)).The substrate was added after these exposure times. In both measurements H_2 (red) was supplied constantly, to keep its concentration constant. The initial styrene concentration was 440 mM. Plots (a) and (b) show the hydrogenation for the catalyst being activated for 5 minutes and 60 minutes, respectively.

IX. Phenomenological relaxation superoperator of H_2 in liquids

Relaxation mechanisms: A ^{1}H nucleus has spin ½ and no chemical shift anisotropy (CSA). Hence, two main intramolecular interactions cause relaxation: dipol-dipol (DD) and spin-rotation (SR). Here, we will consider only the case of extreme narrowing regime, $\omega\tau_C \ll 1$, where ω is the Larmor frequency of the spin and τ_C is the correlation time, because it is fulfilled for H_2 at all available magnetic fields used in MR.

DD mechanism: Under the above mentioned conditions the longitudinal relaxation rate, R_1, and the relaxation rate of the singlet spin state, R_S, for dipolar mechanism are given by[10]:

$$R_1^{\mathrm{DD}} = \left(T_1^{\mathrm{DD}}\right)^{-1} = \frac{3}{2} b_{\mathrm{HH}}^2 \tau_{\mathrm{C}}^{\mathrm{DD}}$$

$$R_{\mathrm{S}}^{\mathrm{DD}} = \left(T_{\mathrm{S}}^{\mathrm{DD}}\right)^{-1} = 0$$

Note that the rate R_{POC} (used in the main text) considers a specific case of singlet relaxation (with rate R_S), i.e. the relaxation of pH$_2$. In the equation above, $b_{\mathrm{HH}} = \frac{\mu_0 \gamma_{1\mathrm{H}}^2 \hbar}{4\pi r_{\mathrm{HH}}^3}$, μ_0 is the vacuum permeability ($\frac{\mu_0}{4\pi} = 10^{-7}\ \mathrm{T}^2 \cdot \mathrm{J}^{-1} \cdot \mathrm{m}^3$), $\gamma_{1\mathrm{H}} = 26.752 \cdot 10^7 \frac{\mathrm{rad}}{\mathrm{s \cdot T}}$ is the magnetogyric ratio of an ^{1}H nucleus and $r_{\mathrm{HH}} = 0.74$ Å is the distance between the two protons of an H$_2$ molecule, $\hbar \cong 1.054 \cdot 10^{-34}\ \mathrm{J \cdot s \cdot rad^{-1}}$ is the Planck constant. Hence, $b_{\mathrm{HH}} = 1.86 \cdot 10^6\ \frac{\mathrm{rad}}{\mathrm{s}}$ and therefore

$$R_1^{\mathrm{DD}} = \left(T_1^{\mathrm{DD}}\right)^{-1} \cong 5.1978 \cdot 10^{12} \mathrm{s}^{-2} \cdot \tau_{\mathrm{C}}^{\mathrm{DD}}$$

Hence, if the T_1 relaxation time of H$_2$ is 1 s and only DD relaxation mechanism is present, it corresponds to the correlation time $\tau_{\mathrm{C}} = \frac{2\ \mathrm{s}^{-1}}{3 b_{\mathrm{HH}}^2} = 0.1922$ ps. In the phenomenological relaxation model presented here the relaxation superoperator is scaled in such a way to achieve the desired (measured) value of T_1 (see below).

Spin-rotation mechanism: H$_2$ belongs to the $D_{\infty h}$ symmetry group. Therefore, the z-component of the moment of inertia is $\mathrm{I}_Z = 0$ as well as zz-principal components of the spin-rotation coupling tensor $C_{zz,1} = C_{zz,2} = 0$. The z principal axis of two spin-rotation tensors are parallel (Euler angle $\Theta = 0$)[10]. Therefore, SR rates for longitudinal relaxation and the lifetime of the singlet spin state are:

$$R_1^{\mathrm{SR}} = \left(T_1^{\mathrm{SR}}\right)^{-1} = 4k \mathrm{C}_{\perp}^2 \mathrm{I}_{\perp} \tau_{\mathrm{C},\perp}^{\mathrm{SR}}$$

$$R_{\mathrm{S}}^{\mathrm{SR}} = \left(T_{\mathrm{S}}^{\mathrm{SR}}\right)^{-1} = 0$$

Here $\mathrm{C}_{\perp,\mathrm{n}}(^1\mathrm{H\ in\ H_2}) \cong 116$ kHz[11], $\mathrm{I}_{\perp} = \frac{m_{1\mathrm{H}} r_{\mathrm{HH}}^2}{4} = 0.2286 \cdot 10^{-47}\ \mathrm{kg \cdot m^2}$ is moment of inertia, $r_{\mathrm{HH}} = 0.74$ Å, $m_{1\mathrm{H}} = 1.67 \cdot 10^{-27}\ \mathrm{kg}$, $k = \frac{k_B T}{3\hbar^2} = 124.22 \cdot 10^{45}\ \mathrm{J^{-1} \cdot s^{-2} \cdot rad^2}$, $k_B = 1.38 \cdot 10^{-23}\ \mathrm{m^2 \cdot kg \cdot s^{-2} \cdot K^{-1}}$ is Boltzmann constant, $T \cong 300$ K, hence

$$R_1^{\mathrm{SR}} = \left(T_1^{\mathrm{SR}}\right)^{-1} = 1.528 \cdot 10^{10}\ \mathrm{s}^{-2} \cdot \tau_{\mathrm{C}\perp}^{\mathrm{SR}}$$

Hence, if $\tau_{\mathrm{C},\perp}^{\mathrm{SR}} \cong 3\tau_{\mathrm{C}}^{\mathrm{DD}}$ the spin-rotation mechanism can be neglected, because its impact to T_1 relaxation is ≈100 times smaller than the contribution from the DD interaction.

Model of uncorrelated local fluctuating magnetic field (LFF): LFF is a commonly used model for simulating T_1 relaxation behavior of multi-spin systems.[12] Since both DD and SR relaxation mechanism do not provide relaxation of the singlet spin state it is necessary to add an additional relaxation mechanism. If the longitudinal relaxation rate of both protons is $R_1^{\mathrm{LFF}} = R_1$ then the relaxation of multispin orders like $\hat{I}_Z \hat{S}_Z$ or the relaxation of singlet spin order is twice as fast, $R_{\mathrm{S}}^{\mathrm{LFF}} = 2R_1$, because in the LFF model spins relax without mutual interaction (fluctuating local field are uncorrelated). Hereafter $\hat{I}_k$ and $\hat{S}_k$ are k-projections (X, Y or Z) of nuclear spin operators of two H$_2$ protons. Therefore, LFF model allows a simple addition of relaxation to the singlet spin state of H$_2$, which is “immune” to the intermolecular relaxation mechanism ($R_{\mathrm{S}}^{\mathrm{DD}} = 0$, see above).

H_2 relaxation superoperator: All required equations to calculate the relaxation superoperators $\hat{\hat{R}}^{\mathrm{DD}}$ and $\hat{\hat{R}}^{\mathrm{LFF}}$ are available in literature[10,13]. The following superposition of two superoperators

$$\hat{\hat{R}}^{\mathrm{H_2}}[T_1, \tau_{\mathrm{POC}}] = \hat{\hat{R}}^{\mathrm{DD}}[0.74\,\mathrm{A}, 0.1922\, ps] \cdot (1/T_1 - 1/2\tau_{\mathrm{POC}}) \cdot 1s + \hat{\hat{R}}^{\mathrm{LFF}}[2\tau_{\mathrm{POC}}] \quad (1)$$

can be used for the simulation of H_2 relaxation. Because from the above described properties of relaxation superoperators it follows

$$R_1^{\mathrm{H_2}} = 1\,\mathrm{s}^{-1} \cdot \left(\frac{1}{T_1} - \frac{1}{2\tau_{\mathrm{POC}}}\right) \cdot 1\,\mathrm{s} + \frac{1}{2\tau_{\mathrm{POC}}} = \frac{1}{T_1}$$

$$R_S^{\mathrm{H_2}} = 0 \cdot \left(\frac{1}{T_1} - \frac{1}{2\tau_{\mathrm{POC}}}\right) \cdot 1\,\mathrm{s} + 2 \cdot \frac{1}{2\tau_{\mathrm{POC}}} = \frac{1}{\tau_{\mathrm{POC}}}$$

that gives the relaxation rates corresponding to T_1 and τ_{POC}.

For a two spin system of H_2 there are three relaxation Eigenstates.[14] This Eigenstates can be represented as (1) net magnetization (relaxation rate R_1), (2) singlet state (relaxation rate R_S) and (3) superposition of the triplet states (in the following referred to as triplet Eigenstate): $\sqrt{2}I_z S_z - \left(I_x S_x + I_y S_y\right) \propto P_{T_+} + P_{T_-} - \sqrt{2}P_{T_0}$, here P_k are the populations of the corresponding triplet spin states (relaxation rate R_T). The relaxation rate of the triplet Eigenstate in the DD and LFF models are $R_{\mathrm{T}}^{\mathrm{DD}} = 3R_1^{\mathrm{DD}}/5$ and $R_{\mathrm{T}}^{\mathrm{LFF}} = 2R_1^{\mathrm{LFF}}$, therefore

$$R_{\mathrm{T}}^{\mathrm{H_2}} = \frac{3}{5} 1s^{-1} \cdot \left(\frac{1}{T_1} - \frac{1}{2\tau_{POC}}\right) \cdot 1s + 2 \cdot \frac{1}{2\tau_{POC}} = \frac{3}{5}\frac{1}{T_1} + \frac{7}{10\tau_{POC}} \xrightarrow{T_1 \ll \tau_{POC}} \frac{3}{5}\frac{1}{T_1}$$

Hence, the triplet Eigenstate relaxes with almost the same rate as it is predicted by the DD mechanism.
Using typical relaxation times for H_2 dissolved in pure solvents (here, T_1 = 1.5 s and τ_{POC} = 1000 s, see Table 1), we demonstrate the first application of the model: the relaxation of polarized H_2 to thermal equilibrium. The model demonstrates that different spin orders relax to the thermal equilibrium with different rates (Fig. S10). This is necessary for quantitative modeling of the outcome of a PHIP experiment, but was, to date, neglected.

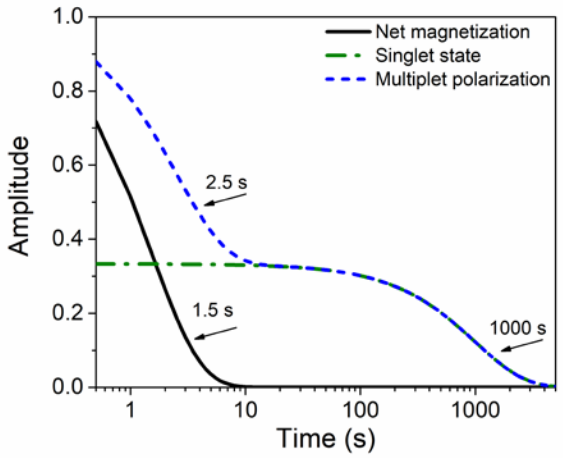

Figure S9. Example of the relaxation kinetics of net magnetization ($\hat{I}_Z + \hat{S}_Z$, black), singlet state ($-\hat{\boldsymbol{I}} \cdot \hat{\boldsymbol{S}}$ green) and multiplet polarization ($\hat{I}_Z\hat{S}_Z$ spin order, blue) of H_2. The initial spin state is a combination of net and multiplet polarizations: $\hat{I}_Z + \hat{S}_Z + \hat{I}_Z\hat{S}_Z$. The assumed input parameters for $\hat{\hat{R}}^{\mathrm{H_2}}$ are $T_1 = 1.5$ s, $\tau_{\mathrm{POC}} = 1000$ s, that correspond to relaxation of net magnetization and the singlet spin state, respectively. Hence, the relaxation time of the triplet Eigenstate is $T_{\mathrm{T}}^{\mathrm{H_2}} = \left(\frac{3}{5}\frac{1}{T_1} + \frac{7}{10}\frac{1}{\tau_{\mathrm{POC}}}\right)^{-1} \cong \frac{3}{5}\frac{1}{T_1} = 2.5\, s$. Note that as the multiplet polarization is not an Eigen state of $\hat{\hat{R}}^{\mathrm{H_2}}$ its behavior is bi-exponential.

References

[1] J.-B. Hoevener, S. Bär, J. Leupold, K. Jenne, D. Leibfritz, J. Hennig, S. B. Duckett, D. von Elverfeldt, *NMR Biomed.* **2013**, *26*, 124–131.
[2] J.-B. Hövener, E. Y. Chekmenev, K. C. Harris, W. H. Perman, L. W. Robertson, B. D. Ross, P. Bhattacharya, *Magn Reson Mater Phy* **2009**, *22*, 111–121.
[3] J.-B. Hövener, E. Y. Chekmenev, K. C. Harris, W. H. Perman, T. T. Tran, B. D. Ross, P. Bhattacharya, *Magn Reson Mater Phy* **2009**, *22*, 123–134.
[4] A. B. Schmidt, S. Berner, W. Schimpf, C. Müller, T. Lickert, N. Schwaderlapp, S. Knecht, J. G. Skinner, A. Dost, P. Rovedo, et al., *Nat. Commun.* **2017**, DOI 10.1038/ncomms14535.
[5] I. D. Gridnev, N. Higashi, K. Asakura, T. Imamoto, *J. Am. Chem. Soc.* **2000**, *122*, 7183–7194.
[6] C. Terenzi, S. Bouguet-Bonnet, D. Canet, *J. Phys. Chem. Lett.* **2015**, *6*, 1611–1615.
[7] G. Banfi, G. L. Salvagno, G. Lippi, *Clin. Chem. Lab. Med.* **2007**, *45*, 565–576.
[8] C. R. Bowers, D. P. Weitekamp, *J. Am. Chem. Soc.* **1987**, *109*, 5541–5542.
[9] M. Haake, J. Natterer, J. Bargon, *J Am Chem Soc* **1996**, *118*, 88–91.
[10] G. Pileio, *Prog. Nucl. Magn. Reson. Spectrosc.* **2010**, *56*, 217–231.
[11] I. A. Aucar, S. S. Gómez, J. I. Melo, C. C. Giribet, M. C. Ruiz de Azúa, *J. Chem. Phys.* **2013**, *138*, 134107.
[12] R. Freeman, S. Wittekoek, R. R. Ernst, *J. Chem. Phys.* **1970**, *52*, 1529–1544.
[13] K. Ivanov, A. Yurkovskaya, H.-M. Vieth, *J. Chem. Phys.* **2008**, *129*, 234513.
[14] A. N. Pravdivtsev, K. L. Ivanov, R. Kaptein, A. V. Yurkovskaya, *Appl. Magn. Reson.* **2013**, *44*, 23–39.
